# Suspect Screening and Prioritization as an Analytical Strategy for the Identification of Persistent, Mobile, and Toxic (PMT) Substances in Surface Water

**DOI:** 10.1021/acs.analchem.5c04907

**Published:** 2026-02-19

**Authors:** Lesly Ayala Cabana, Alejandra Arcas, Isabel López-Heras, Ana de Santiago-Martín, Raffaella Meffe

**Affiliations:** † Geography and Environment Department, Faculty of Sciences, University of Alcalá, Geology, External Campus, Ctra. A-II km 33.6, Alcalá de Henares 28871, Madrid, Spain; ‡ 202527 IMDEA Water, Avda Punto Com, 2, Alcalá de Henares 28805, Madrid, Spain

## Abstract

Persistent, mobile, and toxic (PMT) substances have gained increasing scientific and regulatory attention due to their capacity to bypass natural and artificial barriers and spread throughout the water cycle. However, knowledge of their environmental occurrence remains limited due to analytical challenges, particularly in detecting highly polar substances that are often overlooked in monitoring studies. This study aims to identify PMT substances that are worth monitoring in surface waters strongly influenced by wastewater treatment plant effluents. A suspect screening analysis (SSA) approach based on the use of LC-HRMS was integrated with a tiered prioritization strategy. Our workflow integrates multimodal SPE and LC approaches to improve PMT detection coverage across polarity gradients. A total of 305 substances were tentatively identified, and 103 of them were prioritized as PMT substances, encompassing industrial chemicals, personal care products, pharmaceuticals, illicit drugs, pesticides, and transformation products. Notably, only 13% of PMT substances are currently included in the European Water Framework Directive legislation or the REACH list of substances of very high concern. Among them, 35 high-priority PMT substances were confirmed with analytical standards through mass spectrometry (MS/MS) in tandem with HRMS, providing reliable fragmentation data. Some of these substances such as the pharmaceutical celecoxib, the ultrashort-chain per- and polyfluoroalkyl substance (PFAS) bis­(trifluoromethylsulfonyl)­imide, or the industrial chemical 1,3-di-*o*-tolylguanidine (DTG) have been scarcely investigated in environmental monitoring efforts. The methodological framework presented in this study is readily adaptable to a wide range of environmental scenarios. The results obtained highlight the importance of integrating SSA as a complementary approach to conventional target analysis.

## Graphical abstract

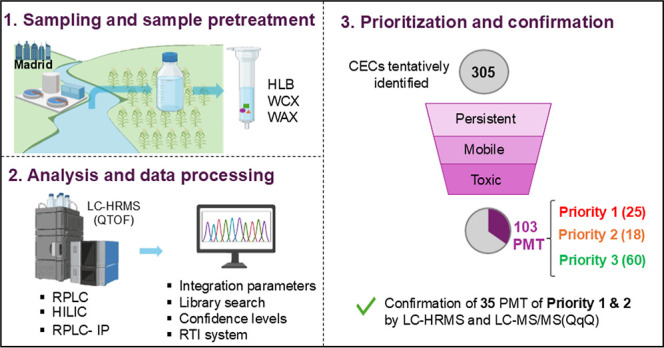

## Introduction

The so-called “chemical universe” includes more than 290 million unique chemical substances (CAS registry). Hundreds of thousands of these substances are registered for production and use, leading to their continuous release into the environment as contaminants of emerging concern (CECs).^1^ Environmental quality standards for CECs are not defined, and their direct and indirect effects on human health are not yet understood.^2^ The sheer number of chemicals hinders comprehensive analytical strategies. In recent years, greater attention has grown toward a subclass of CECs identified as persistent, mobile, and toxic (PMT) substances for their capacity to spread throughout the water cycle and their higher potential of representing a threat to the ecosystems and, through them, to human health.^3^ In 2023, the amended CLP Regulation (Classification, Labeling and Packaging, Regulation (EC) No 1272/2008) introduced the PMT/vPvM (very persistent and very mobile) hazard class for all chemical substances and mixtures under REACH Regulation (Registration, Evaluation, Authorization and Restriction of Chemicals, Reg. (EC) 1907/2006).^4^ This represents a significant milestone in achieving harmonized hazard classification. However, substances not covered by REACH will not be included in this classification, likely leading to an underestimation of the number of PMT substances. Moreover, despite the recognized importance of PMT substances, there is a paucity of information about their presence in the environment related to the limitations of analytical techniques. Conventional analytical approaches have a limited coverage of PMT substances across polarity and charge states.^5,6^ For this reason, a structured suspect screening strategy is essential to efficiently drive research actions.

In this context, different analysis strategies based on high-resolution mass spectrometry (HRMS) and tandem mass spectrometry (MS/MS) techniques have been proposed. The LC-HRMS systems with time-of-flight (TOF), quadrupole-time-of-flight (QTOF), and quadrupole-Orbitrap (Q-Orbitrap) analyzers have demonstrated an excellent ability to detect and identify a broad range of analytes.^7^ However, appropriate sample treatment procedures and tailored separation techniques are critical prerequisites for the successful detection of PMT substances.^8^ One of the major challenges in the suspect screening analysis (SSA) of PMT substances in environmental matrices involves sample preparation, which must ensure sufficient analyte retention and purification selectivity to minimize matrix interferences while maintaining broad analyte coverage and adequate sensitivity.^9,10^ In this sense, solid-phase extraction (SPE) using different sorbents is widely employed in tentative identification studies.^7^ Among SPE materials, hydrophilic lipophilic balanced (HLB) sorbents are frequently employed due to their extensive retention capabilities; however, they are not optimized for charged or ionizable substances. To deal with this analytical gap, ion-exchange SPE materials such as weak anion exchange (WAX) and weak cation exchange (WCX) offer promising alternatives.^3^ Regarding chromatographic conditions, reverse-phase (RP) columns are commonly employed in SSA. Despite their robustness and versatility, the methods based on RP chromatography exhibit limited retention for highly hydrophilic substances.^11^ To overcome this limitation and enable the detection of very polar PMT substances, alternative chromatographic techniques, such as hydrophilic interaction liquid chromatography (HILIC), have been proposed.^12,13^ Similarly, the use of ion-paring (IP) agents such as heptafluorobutyric acid (HFBA) in the mobile phase represents an additional alternative that has been shown to enhance peak shape and retention of very polar substances such as metformin or melamine when using conventional C18 columns.^14,15^

In general, SSAs often result in the tentative detection of a large number of substances. Accordingly, applying prioritization strategies is essential to efficiently focus limited analytical capabilities toward monitoring the most relevant substances.^16^ The hazard quotient (HQ) model remains the most widely used prioritization approach. However, it considers only occurrence and toxicity, neglecting critical parameters such as persistence^17^ or polarity.^18^ Moreover, a significant limitation of these evaluations is the paucity of experimental data in terms of persistence and ecotoxicity of a great proportion of substances.^19^ To deal with this, recent studies have proposed prioritization frameworks for PMT substances based on the combination of experimental data and quantitative structure–activity relationship (QSAR) models.^20,21^

The application of screening workflows addressing the limitations in extraction, separation, and identification combined with an appropriate prioritization strategy of tentatively identified substances is crucial for the effective detection and evaluation of PMT occurrence. This study aims to develop and apply an integrative LC-HRMS-based SSA and prioritization workflow to identify and confirm the presence of PMT substances in wastewater-impacted surface water. The strategy encompasses a comparative evaluation of three SPE sorbents and four LC analysis conditions for PMT detection with a particular emphasis on highly polar substances to address existing analytical and monitoring gaps. Moreover, the implementation of a multipurpose strategy will furnish analytical information that will support the development of a multiresidue quantitative method targeting selected PMTs for future monitoring in different environmental matrices.

## Materials and Methods

The SSA workflow proposed in this study (Figure 1) integrates multiple complementary strategies and comprises five key steps: (i) sample treatment using different extraction conditions and assessment of matrix effects (ME) resulting from substance preconcentration; (ii) SSA by LC-HRMS using RP, ion-pairing reverse-phase (IP-RP) mechanisms, and the HILIC approach for searching highly polar substances; (iii) data processing, (iv) substance prioritization, and (v) confirmation.

**Figure 1:**
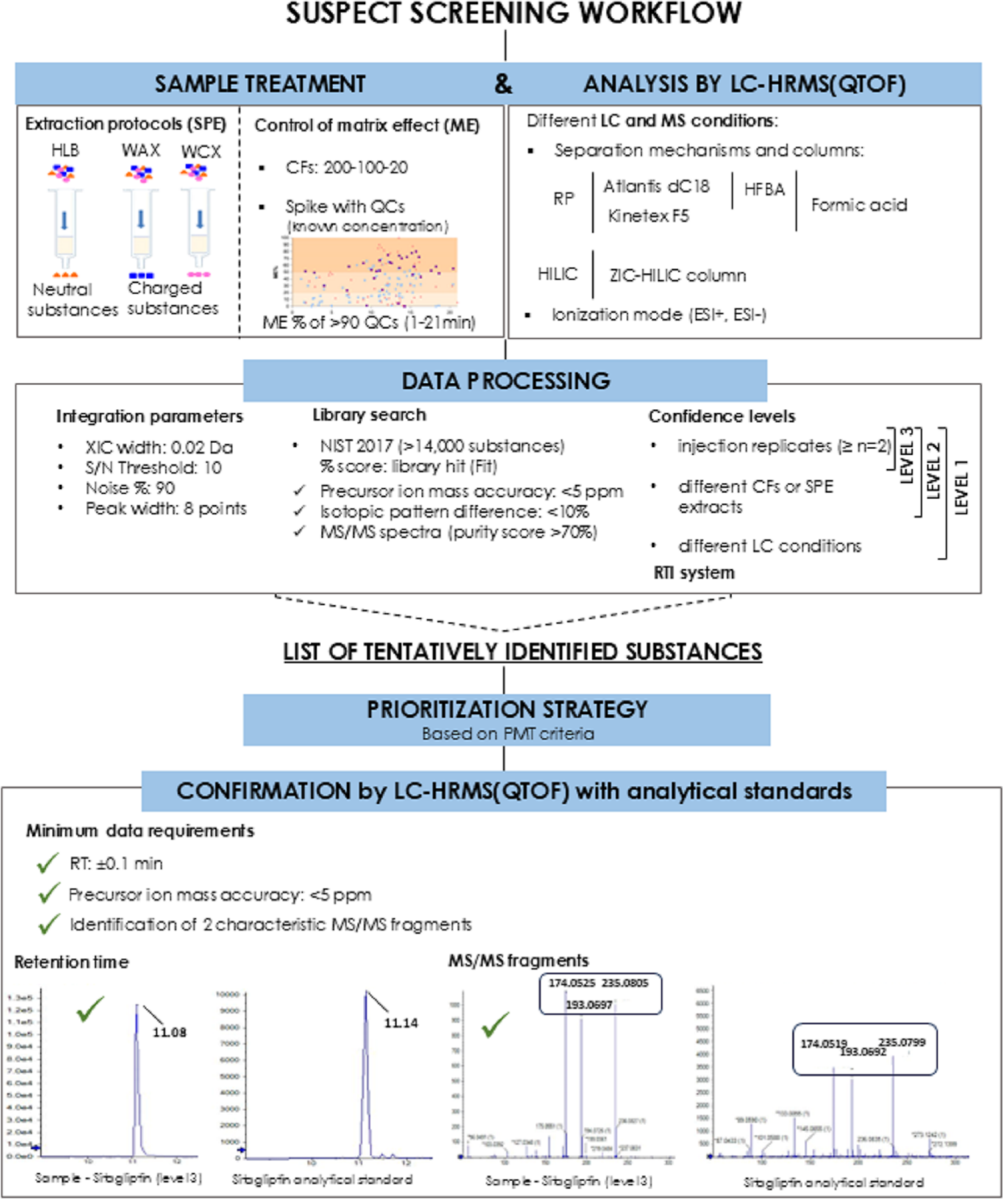
Suspect screening workflow outlining the steps followed for the identification of persistent, mobile, and toxic (PMT) substances: (i) sample treatment, (ii) analysis by LC-HRMS, (iii) data processing, (iv) prioritization, and (v) confirmation.

### Chemicals and Standards

Formic acid (purity ≥98%) and heptafluorobutyric acid (HFBA, 98%) were purchased from Merck (Darmstadt, Germany). Both LC–MS-grade acetonitrile (ACN) and methanol (MeOH) and ammonium hydroxide (NH_4_OH, 32%) were purchased from Scharlau (Barcelona, Spain). Ammonium acetate (CH_3_CO_2_NH_4_, purity >96%) was supplied by Scharlau (Barcelona, Spain), and ammonium formate (HCOONH_4_, purity ≥99%) was purchased from Merck. Ultrapure water was obtained using a Milli-Q purification system (Merck Millipore, Milford, MA, USA).

All analytical standards (purity ≥98%) used in the SSA were purchased from different suppliers (see Table S1 in Supporting Information). These standards were classified into three categories: (i) 98 Quality Controls (QCs) to guarantee the accuracy of screening analyses (resolution and sensitivity); (ii) 11 polar standards to test the chromatographic separation conditions by the HILIC column; and (iii) 18 retention time index (RTI) calibrants to test the application of the RTI approach. The standards (purity ≥98%) for the confirmation of tentatively identified substances (Table S7) were purchased from Merck (Darmstadt, Germany) and Cymit Qumica (Barcelona, Spain). Individual stocks of all analytical standards at 1000 or 2000 mg/L were prepared in MeOH and stored in amber glass vials at −20 °C. Working solutions were prepared by the appropriate mixture and dilution of stock solutions in MeOH/water 10:90% (v/v).

### Sampling and Sample Treatment

The surface water used for the SSA and prioritization was collected in April 2022 from the head of the “Real Acequia del Jarama” (RAJ) channel system (southeastern region of Madrid, Spain). Upgradient of the sampling point, the water receives the effluents of the major wastewater treatment plants (WWTPs) in the Spanish capital. Therefore, an elevated amount of PMT substances is expected to occur. In previous studies, we observed a stable chemical fingerprint over time, confirming that the collected sample can be considered as representative of the water quality.^22^ The water sample was collected using amber glass bottles with poly­(tetrafluoroethylene) (PTFE) caps. In situ measurements included pH (7.7), electrical conductivity (800 μS/cm), dissolved oxygen (6.94 mg/L), and redox potential (−18.5 mV). After retrieval, the sample was immediately transported under refrigerated conditions to IMDEA Water laboratories and stored at −20 °C until analysis.

For the extraction of PMT substances, the sample was thawed overnight in the fume hood and subsequently homogenized prior to filtration through a 0.7 μm glass fiber filter (Merck Millipore, Cork, IRL). Then, sample aliquots (200 mL) were submitted to three different SPE protocols adapted from Montes et al. (2019) to extract neutral, anionic, and cationic PMT substances.^23^ These protocols employed polymeric sorbents (Oasis HLB) and two ionic exchange cartridges (Oasis WAX and Oasis WCX). Each protocol employed specific conditioning, wash, and elution steps, as detailed in Supporting Information 1.2. Eluted fractions from different SPE protocols were evaporated to dryness in a Speed Vac concentrator (ThermoScientific, USA) at 45 °C and 0.9 Torr. Then, the extracts were reconstituted in 1 mL of MeOH/water 10:90% (v/v) and centrifuged for 5 min at 13,000 rpm in a MiniSpin centrifuge (Eppendorf, USA). The SPE extracts at a concentration factor of 200 (CF 200) were diluted 2 and 10 times (CF 100 and CF 20, respectively) and finally transferred to an amber glass vial before LC-HRMS analysis.

In addition, to test the ME, aliquots (450 μL) of CF 200, CF 100, and CF20 SPE extracts from the cartridge able to retain the highest number of compounds were mixed with 50 μL of a 50 μg/L solution containing 98 QCs (83 substances ionizing in positive electrospray ionization (ESI+) and 15 in negative electrospray ionization (ESI−)) to evaluate the effect of preconcentrated matrix interferences on the analyte ionization response. This response was determined as ME% by comparing the peak area of the QCs in fortified SPE extract (at CF 200, CF 100, and CF 20) with the peak area of QCs in MeOH/water 10:90% (v/v) media.

### Analysis by Liquid Chromatography Coupled to High-Resolution Mass Spectrometry

An LC system (1260 series, Agilent Technologies, Palo Alto, CA, USA) coupled with an ESI interface (DuoSpray Ion Source, SCIEX, Framingham, MA, USA) to a quadrupole time-of-flight (QTOF) analyzer (triple TOF 5600 SCIEX) was used for SSA. The source parameters were as follows: a capillary temperature of 550 °C; a nebulizer and drying N_2_ gas pressure of 55 and 30 psi, respectively; an ion spray floating voltage of 5500 and −4500 V (in ESI+ and ESI–, respectively); and an accumulation time of 0.5 s. SWATH (Sequential Window Acquisition of all Theoretical Mass Spectra) fragmentation of SCIEX was applied to the acquisition of full-scan MS over the range of 50–1000 *m*/*z* (with an accurate mass of less than 5 ppm and a resolution of 30,000 fwhm), enabling the collection of MS/MS spectra each 25 amu with a collision energy (CE) of 35 ± 15 V^24^. Calibration was done every 6 injections with positive and negative calibration solutions for the SCIEX Triple TOF systems, which include 12 components with masses between 144.1030 and 1521.9715 Da. Data acquisition was performed by using Analyst TF software.

Different LC strategies were applied to analyze all SPE extracts and blanks obtained using the extraction protocols described in Supporting Information 1.2.

### Reverse-Phase Liquid Chromatography

Two RP columns were used for the SSA: (i) Atlantis dC18 (Waters, Milford, MA, USA), a silica-based column used for the retention of polar and nonpolar substances, and (ii) Kinetex F5 (Phenomenex, Torrance, CA), a pentafluorophenyl propyl column that provides a very high degree of steric selectivity to separate structural isomers and cationic substances. Both columns had the same dimensions (150 mm × 2.1 mm inner diameter) and contained 3.0 μm particles. Guard columns (5 mm × 2.1 mm inner diameter) of the same compositions as each stationary phase were also employed. Mobile-phase compositions for both columns in positive and negative ionization mode, as well as other separation parameters (gradient, flow, column temperature, and injection volume), are detailed in Supporting Information 1.3.

An additional RPLC mechanism using the Atlantis dC18 column with a mobile phase containing HFBA as IP (chromatographic parameters are described in Supporting Information 1.3) was performed together with hydrophilic interaction liquid chromatography for the identification of more polar PMT substances.

### Hydrophilic Interaction Liquid Chromatography

The HILIC column used was a SeQuant ZIC-HILIC (150 × 2.1 mm i.d.; particle size 3.5 μm, Merck) based on a silica sorbent bonded to a stationary phase consisting of a highly polar, permanent zwitterion. Before the screening, separation conditions and the retention efficiency of the column were evaluated by the injection of a group of 10 polar standards (Table S1) and the application of different separation gradients (details are summarized in Table S4). In addition, these analytical standards were also injected into the other chromatographic columns to evaluate their presence in the sample. According to the obtained results in terms of acceptable peak shape, resolution, and reproducibility, an initial composition of 90% of eluent A (ACN/water with 5 mM of ammonium formate 90:10% (v/v)) and the subsequent decrease to 50% in 15 min provides the best separation of standards using a ZIC-HILIC column. The composition of the mobile phases in positive and negative ionization mode as well as other separation parameters (gradient, flow, column temperature, and injection volume) are detailed in Supporting Information 1.4.

### Data Processing

Data were processed with SCIEX OS-Q 1.5 software. Integration parameters were selected as follows: (i) minimum peak width/height: 8/500; (ii) peak-to-peak baseline noise: 10; (iii) mass tolerance (mass window width): 0.02 Da; and (iv) Gaussian smooth width: 2 points. The acquired MS/MS patterns were automatically searched by spectral comparison using the National Institute of Standards and Technology (NIST) 2017 Spectral Library 1.0.1, which contains *m*/*z* values and spectral information on more than 14,000 substances. Library score parameters were selected as follows: (i) results shortened by Fit (using only peaks occurring in the library spectrum) and (ii) a precursor mass tolerance of 0.4 Da.

The criteria for tentatively identifying substances were based on the approach described by Rico et al. (2019),^25^ which includes accurate mass error of precursor ion (mass accuracy <5 ppm), isotopic pattern match (deviation <10%), and automatic MS/MS spectral library matching (library score >70%) considering the most characteristic fragments (abundance and nominal *m*/*z*). Besides filtering by MS and MS/MS criteria, the frequency of detection of *m*/*z* candidates in analysis replicates (*n* = 3) and under different experimental conditions was also used to provide an additional degree of confidence in the tentative identification. Three confidence levels, from level 3 (more tentative identification) to level 1 (less tentative identification), were defined based exclusively on the frequency of detection of a substance under the different analysis methodologies (SPE protocols, concentration factors, and chromatographic separations):

•Level 1: substances detected in at least two analysis replicates (*n* ≥ 2) of a SPE extract (HLB, WAX, WCX) at two CF (100 and 20) and by at least two LC columns (Atlantis dC18, Kinetex F5, and ZIC-HILIC).
•Level 2: substances detected in analysis replicates (*n* ≥ 2) of an SPE extract (HLB, WAX, WCX) at two CF (100 and 20) or by at least two LC columns (Atlantis dC18, Kinetex F5, and ZIC-HILIC)
•Level 3: substances detected in analysis replicates (*n* ≥ 2) of an SPE extract (HLB, WAX, WCX) at one CF (100 or 20) by one LC column (Atlantis dC18 or Kinetex F5 or ZIC-HILIC).

To evaluate an additional level of confidence in the confirmation step, the Retention Time Index (RTI) approach proposed by Aalizadeh et al. (2021) was applied. In this context, 18 RTI calibrants (Table S1), at a concentration ranging from 50 to 500 μg/L, were analyzed by LC-HRMS under the same analytical conditions used for the Atlantis dC18 column in ESI+, as described in Supporting Information 1.3. The linearity and sensitivity of the experimental RTI system were assessed by injecting the calibrants both in a MeOH/water 10:90% (v/v) medium and in the preconcentrated extract obtained from the three SPE cartridges (HLB, WCX, and WAX). The calibration curves and RTI predictions were generated using the RTI model available at http://rti.chem.uoa.gr/.^26^ The predicted RTI values were then applied to the substances selected for the confirmation step (see Confirmation Section).

### Prioritization Strategy

Based on the defined criteria of interest, a four-step prioritization methodology (Figure 2) was implemented to classify and select the substances for confirmation using reference standards. The PMT classification was developed in previous studies,^20,21,27^ while the priority levels were defined in this study. In this work, the set of databases consulted for retrieving data has been extended to include NORMAN EMPODAT (https://www.norman-network.com/nds/), IPChemPortal (https://ipchem.jrc.ec.europa.eu/), Gov.UK (https://www.data.gov.uk/), UCMR-Unregulated Contaminant Monitoring Rule of EPA (https://www.epa.gov/dwucmr), the ED List (https://edlists.org/the-ed-lists), and TEDX List (https://endocrinedisruption.org/).

**Figure 2:**
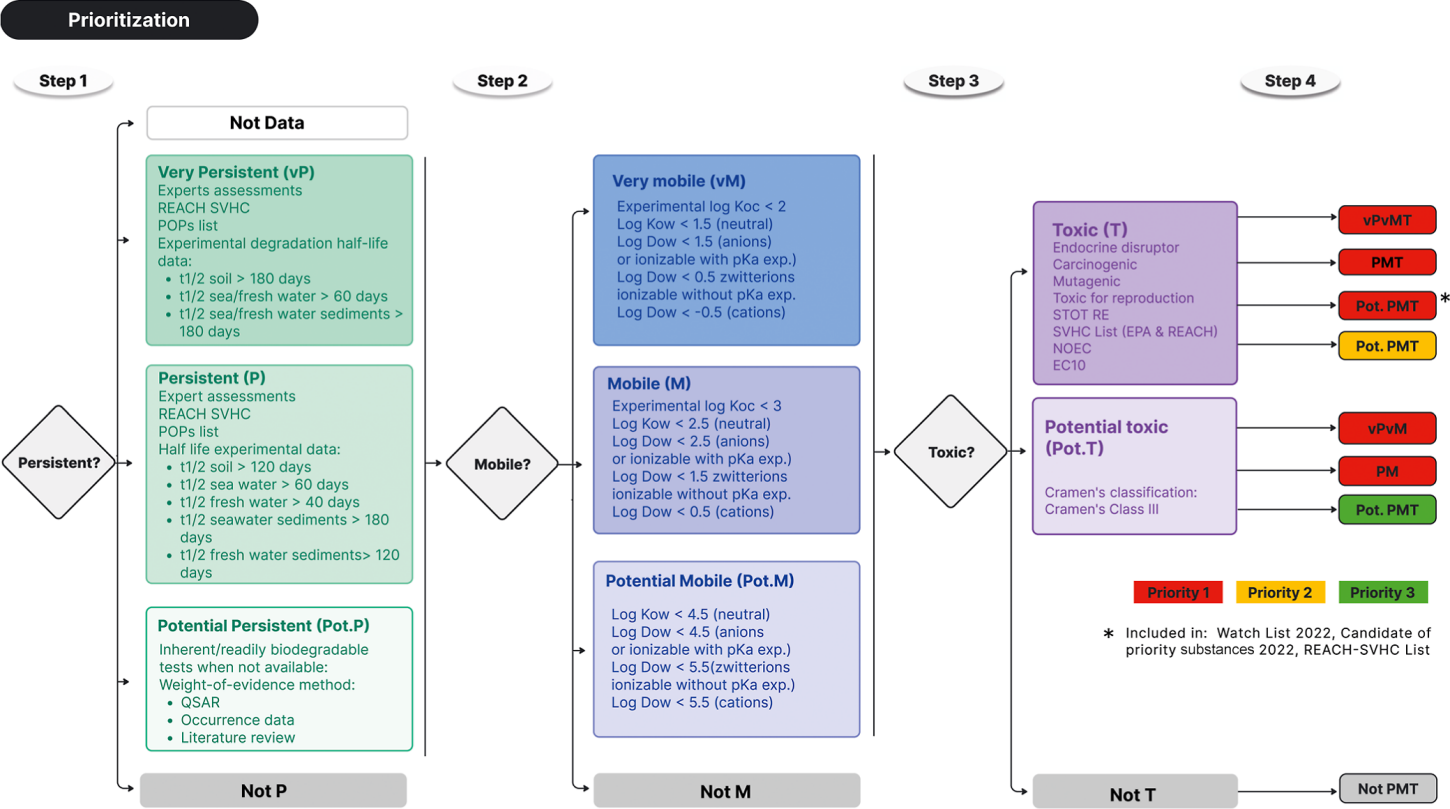
Overview of the prioritization strategy for selecting substances of interest based on persistence, mobility, and toxicity properties.

Step 1. The persistence of the tentatively identified substances was assessed by defining five main categories: Very Persistent (vP), Persistent (P), and Potential Persistent (Pot. P), Not Persistent (Not P), and, in the case of insufficient or conflicting data, the substances were designated as belonging to the No Data category. Substances classified as P or vP by experts^20,21,27^ or those already included in the REACH list of substances of very high concern (REACH-SVHC) as PBT/vPvB substances (where B stands for bioaccumulative) or included in the Stockholm Convention’s Persistent Organic Pollutants (POPs) list were automatically assigned to the P or vP category. For the remaining substances, simulated half-life values (*t*_1/2_) obtained from the EchemPortal database were evaluated following the criteria established in Annex XIII of REACH for substances characterized as P and vP (ECHA, 2017).^28^ Subsequently, the same database was queried for inherently or readily biodegradable tests to screen for Not P or Pot. P. For substances lacking experimental data, a Weight of Evidence (WOE) approach was employed, integrating diverse quantitative structure–activity relationship (QSAR) models and occurrence data. The P prediction tool within the QSAR Toolbox, OPEn Structure–Activity/Property Relationship App (OPERA), and the EPI Suite BIOWIN models from 1 to 6 (acquired through QSAR Toolbox) were used. Both the European Chemicals Agency (ECHA) criteria for PBT/vPvB substances and the regression models by Arnot et al. (2005)^100^ were applied to obtain freshwater *t*_1/2_ values from the BIOWIN model. Furthermore, occurrence data of these substances in drinking water and/or groundwater were collected from the above-mentioned databases (NORMAN EMPODAT, IPChemPortal, Gov. UK, UCMR-Unregulated Contaminant Monitoring Rule of EPA). The presence of these substances in these compartments serves as an indicator of their persistence as it means that they pass through natural and artificial barriers. Substances fulfilling the persistence criterion only through the WOE approach were classified as Pot. P.

Step 2. Substances with different degrees of persistence were further classified by considering their mobility into three main categories: Very Mobile (vM), Mobile (M), and Not Mobile (Not M). The classification adheres to the criteria proposed by CLP Regulation for M/vM substances (log K_oc_ < 3/<2). The experimental values of logK_oc_ obtained from the EchemPortal (https://www.echemportal.org) and UFZ-LSER-R databases (https://www.ufz.de/lserd) were utilized. For substances for which no experimental data were found, the Chemicalize platform was used to calculate logK_ow_ values.

Step 3. Toxicity assessment was conducted following the criteria outlined in Annex XIII of REACH (ECHA, 2017).^28^ To adopt a conservative approach, additional categories and complementary classifications were utilized, as proposed by Neuman et al. (2019).^21^ Initially, a search was conducted on the ECHA website for substances classified as carcinogenic (cat. 1A, 1B, 2), germ cell mutagenic (cat. 1A, 1B, 2), toxic for reproduction (cat. 1A, 1B, 2), or specific organ systemic toxicity upon repeated exposure (STOT RE) (cat. I, II). Additionally, values for the No-effect concentration (NOEC) and 10% effect concentration (EC10) for marine and freshwater organisms were obtained from the EnviroTox database (https://envirotoxdatabase.org). Substances were also classified based on their potential endocrine-disrupting properties, utilizing information from the ECHA’s endocrine disruptor assessment list (https://echa.europa.eu/es/ed-assessment), CHEMSec SIN List (https://sinlist.chemsec.org), and TEDX List. In cases where substances could not be classified according to the previous criteria, the Cramer classes obtained from the QSAR Toolbox software were used to classify the substances with class III as Potential Toxic (Pot. T), while those belonging to classes I and II were categorized as nontoxic (Not T).

Step 4. According to the classification described so far based on the P, M, and T criteria, substances were categorized into the following six classes (Figure 2):

1. vPvM: substances designated as vP and vM. This category also includes vM and Pot. P substances with additional WOE data about their persistence.
2. PM: substances categorized as P and M but not as T.
3. PMT: substances categorized as P, M, and T.
4. vPvMT: substances designated as vP and vM and additionally categorized as T.
5. Pot. PMT: substances with Pot P and M/vM, lacking additional WOE data about their persistence but classified as T or Potential T.
6. Not PMT: substances classified as not P or not M.

Finally, the categorization of PMT substances was carried out using three different priority levels:

- Priority 1: substances classified as PM, PMT, vPvM, vPvMT, as well as Pot. PMT included in the 2022 Watch List and/or a candidate in the List of Priority Substances 2022 (EC, 2022)^29^ and/or included in the REACH-SVHC List.
- Priority 2: potential PMT classified as T.
- Priority 3: potential PMT classified as Potential T.

Henceforth, the term “PMT substances” refers to substances classified in this study into one of five classes in step 4, including vPvM, PM, PMT, vPvMT, and Pot. PMT.

### Confirmation Assay

Tentatively identified PMT substances (priority 1 and 2) were confirmed by coinjection of the SPE extracts with analytical standard stock solutions under the SSA conditions described above. The RT and MS/MS spectra of pure standards were compared to experimental data obtained from the SPE extracts. When confirmation by LC-HRMS was not conclusive, analytical standards and SPE extracts were analyzed using an LC system (1290 series, Agilent Technologies, Palo Alto, CA, USA) coupled to a triple quadrupole (QqQ) mass spectrometer (6495C, Agilent Technologies) to achieve unequivocal confirmation. The MS/MS analyses were performed using the ESI parameters established by Huidobro-López et al. (2023)^30^ (Supporting Information 1.1.).

## Results and Discussion

### Suspect Screening Analysis of Surface Water

#### Sample Pretreatment and Control of the Matrix Effect

The three SPE protocols applied to achieve a compromise between selectivity and sensitivity without bias against negatively and positively charged polar molecules led to the tentative identification of 302 substances. The HLB protocol retains the highest number of substances (184) through both hydrophilic and lipophilic interactions, while 153 and 124 substances are extracted by WCX and WAX, respectively. Among the 302 substances, 72 are retained by all three protocols. On the other hand, 50 substances are uniquely extracted using an HLB cartridge, while WCX and WAX selectively retain 40 and 19 substances, respectively. At pH 7, the ionization state distribution of substances is diverse: 40% neutral, 23% cationic, 22% anionic, 11% ionizable, and 4% zwitterionic. These results highlight the advantage of combining multiple SPE protocols to enable the extraction of a broad spectrum of substances, bridging the gap between permanently charged and ionizable substances.

After the extraction protocol is applied, matrix components in the water sample are preconcentrated along with analytes of interest, which often results in the suppression of their ionization response and the inaccurate interpretation of data. In this study, the complexity of the water sample due to the strong inputs from wastewater effluents prompted us to investigate possible ME on extracted and preconcentrated analytes. The ME is controlled by applying different strategies such as the use of internal standards, additional cleaning steps, or dilution.^31^ In the present work, HLB sample extracts were used to evaluate the ME, as these cartridges allow the identification of a higher number of compounds and produce better peak shapes compared with WCX and WAX cartridges. The selected QCs (Table S1) are chemical standards with different structural characteristics, polarities, and elution times (between 1.65 and 21 min) in RPLC. As an example, Figure 3 shows the ME% of detected QCs (approximately 94% of the ESI + QCs) plotted against their t_R_ by using the Kinetex F5 column. A ME < 20% is considered low, between 20% and 50% is assumed to be moderate, whereas values >50% indicate the presence of strong matrix effects.^32^ Approximately 43% of the QCs show ME <20%, while 35% display a moderate ME (20–50%). In CF 200, the lowest number of QC substances was detected, with only 47% detected compared to detection rates above 66% and 75% in CF 100 and CF 20, respectively. Moreover, as shown in Figure 3, 67% of the substances detected in CF 200 show a ME > 50%. The ME is minimized for most substances in more diluted SPE extracts (CF 100 and 20). Moreover, the number of QCs eluted between 1 and 5 min is much lower (only three are detected) at CF 200 in comparison with those detected at CFs 100 or 20 (∼20). These results are likely attributed to the higher presence of interferences at CF 200 that coelute with more polar substances during the early stages of the RPLC separation.^33^ A similar trend is observed with the Atlantis dC18 column (data not shown). Based on these findings, data processing of the more concentrated SPE extract (CF 200) was discarded, and the tentative identification was undertaken exclusively in CF 20 and CF 100 of HLB, WCX, and WAX SPE extracts. Such a strategy allows us to reduce the time of processing while gaining more accuracy in data treatment. This analytical methodology is fully applicable to other types of water samples as long as potential matrix interferences are considered.

**Figure 3:**
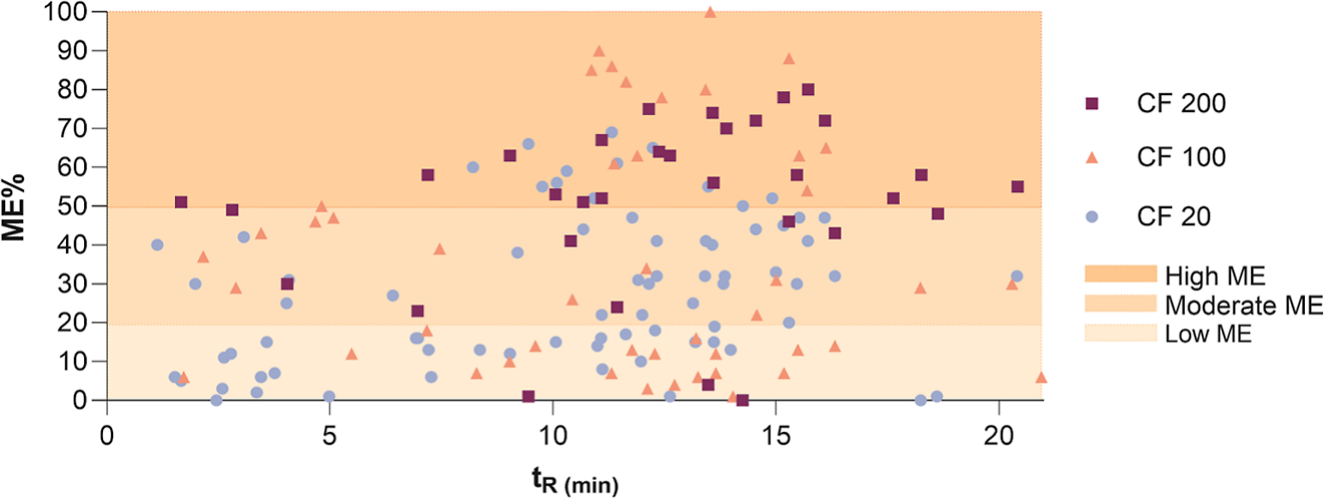
Matrix effect percentage (ME%) and retention time (*t*_R_) of QCs in HLB sample extracts at CF 200, 100, and 20 by LC-HRMS analysis using a Kinetex F5 column.

#### Search for PMT Substances through Different Chromatographic Methods

The separation and analysis of environmental samples containing a complex mixture of analytes across a wide polarity range remain as a significant analytical challenge. To address this, four chromatographic conditions were employed. Regarding RPLC methods using Atlantis dC18 and Kinetex F5 columns with formic acid as the modifier, 227 substances meet the established MS and MS/MS tolerance criteria and detection frequency thresholds (corresponding to confidence levels from 1 to 3). Among these, 43 and 51 substances are uniquely detected by Atlantis dC18 and by Kinetex F5, respectively, and 98 substances are common to both columns (Figure 4a). The use of Atlantis dC18-HFBA (as IP-RPLC) and ZIC-HILIC mechanisms allows the detection of 31 and 43 additional substances, respectively. The use of HFBA as an IP agent in RPLC enhances the chromatographic performance of polar substances by a substantial increase of t_R._ The t_R_ using HFBA is higher than 5 min for all detected substances (Figure S1c). Similar results have been reported by Al-Odaini et al. (2010).^14^ When comparing both methods using the same stationary phase (Atlantis dC18), the HFBA method allows the detection of 49 extra substances. Moreover, only 21 substances (∼7% of the total) are commonly detected under RPLC, IP-RPLC, and HILIC conditions, underscoring the complementarity of these methods and highlighting the importance of employing multiple chromatographic strategies in SSA.

**Figure 4:**
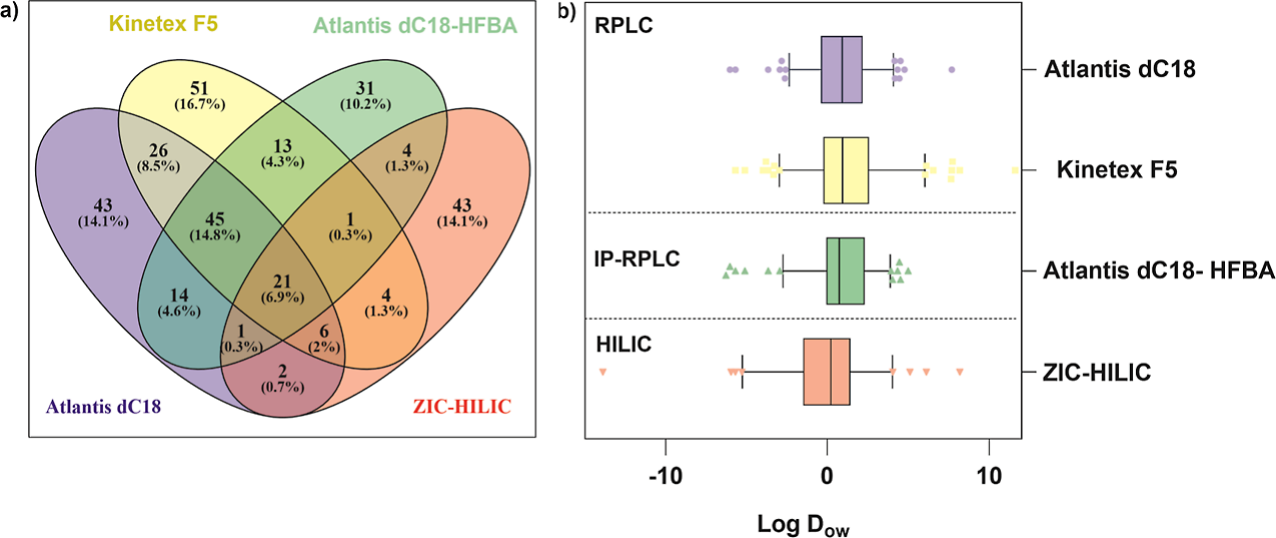
(a) Overview of the number of tentatively identified compounds using Atlantis dC18, Kinetex F5, Atlantis dC18-HFBA, and HILIC (confidence level from 1 to 3). Oliveros, J.C. (2007–2015) Venny (https://bioinfogp.cnb.csic.es/tools/venny/index.html). (b). Box plots showing the distribution of the log *D*_ow_ (pH 7) of the tentatively identified substances according to the chromatographic mode.

Regarding polarity, RPLC mechanisms are known to be suitable to retain and separate analytes from nonpolar to moderately polar molecules (log *D*_ow_ > 0)^34^. Comprehensive screening of the more polar PMT substances (log *D*_ow_ < 0) is accomplished by utilizing the HILIC mechanism and by substituting the formic acid with HFBA as the modifier in RP chromatographic separation. Comparison of the substances detected under different chromatographic conditions (Figure 4b) revealed significant differences (*p* < 0.05) in the polarity of the substances tentatively detected by the ZIC-HILIC column and the other chromatographic methods. Although HFBA improves chromatographic quality and facilitates the detection of certain additional substances, it does not enhance the detection of additional highly polar substances. However, HILIC successfully addresses a significant analytical gap by enabling the detection of 21 very hydrophilic substances with negative log D_ow_ values at pH 7, for which the use of RPLC columns is typically ineffective.^13^ In addition, the application of this analytical method allows us to detect other 20 substances considered as mobile according to the classification criteria (log *D*_ow_ < 3).

There is a significant Pearson correlation (*p* < 0.05) between *t*_R_ and log *D*_ow_ for all separation approaches. However, this correlation is weaker for the HFBA and HILIC methods compared with Atlantis dC18 and Kinetex F5 (Figure S1). In the case of HILIC, this reduced correlation can be attributed to the more complex retention mechanisms involved, which include ion-exchange and electrostatic interactions in addition to partitioning.^35^ As expected, RPLC shows early elution of polar substances, whereas in HILIC, nonpolar substances elute earlier.^34,36^

As mentioned in Hydrophilic Interaction Liquid Chromatography Section, 10 analytical standards were injected into the different chromatographic columns. This targeted analysis enables the additional confirmation of three substances (Table S1) that were not tentatively detected by SSA but were added to the 302 substances initially identified. A key limitation of SSA when using libraries lies in the spectral variability induced by differences in instrumentation, MEs, or low analyte concentrations.^37^ These factors may compromise the accuracy of the spectral matching, resulting in false negatives.

In summary, 305 substances characterized by a broad range of physicochemical properties were tentatively identified in a complex environmental sample using our workflow, which combines different SPE protocols with polarity-extended chromatographic conditions. These identifications serve as a basis for subsequent prioritization and confirmation purposes. Further details on the results of the SSA (compound name, formula, t_R_, molecular ion, signal-to-noise ratio (S/N), mass accuracy, library score, SPE extract, and confidence level) are provided in Table S2 of the Supporting Information.

#### Prioritization

The 305 substances tentatively identified in the surface water sample were classified according to their use in the following classes: (i) industrial chemicals, (ii) personal care products (PCPs), (iii) active pharmaceutical ingredients (APIs), (iv) pharmaceutical reagents/intermediates, (v) transformation products (TPs) of pharmaceuticals, (vi) illicit drugs, (vii) pesticides, and (viii) pesticide TPs (Figure 5). The category of industrial chemicals covers multiple uses in many different fields of application such as coating products, production of adhesives, products used in polymerization processes, etc. Most of the detected substances are pharmaceuticals, a finding consistent with previous studies reporting their prevalence in the water cycle.^11^ Among all substances, 38 are identified as natural products with no known uses reported in the consulted databases. Additionally, two identified herbicides (diuron and terbutryn) are excluded from further evaluations as they do not meet the criteria to be defined as CECs (i.e., they are included in the List of Priority Substances (Directive 2013/39/EU). It is noteworthy that 70% of detected CECs are not registered under the REACH regulation or harmonized in Annex VI of the CLP Regulation. This regulatory gap suggests that there are a considerable number of substances present in the environment that may pose an unknown or unassessed risk. Also, 49% of total detected substances (of which 26% are pharmaceutical TPs) have not been previously evaluated for their PMT properties in expert assessments.^20,21,27^

**Figure 5:**
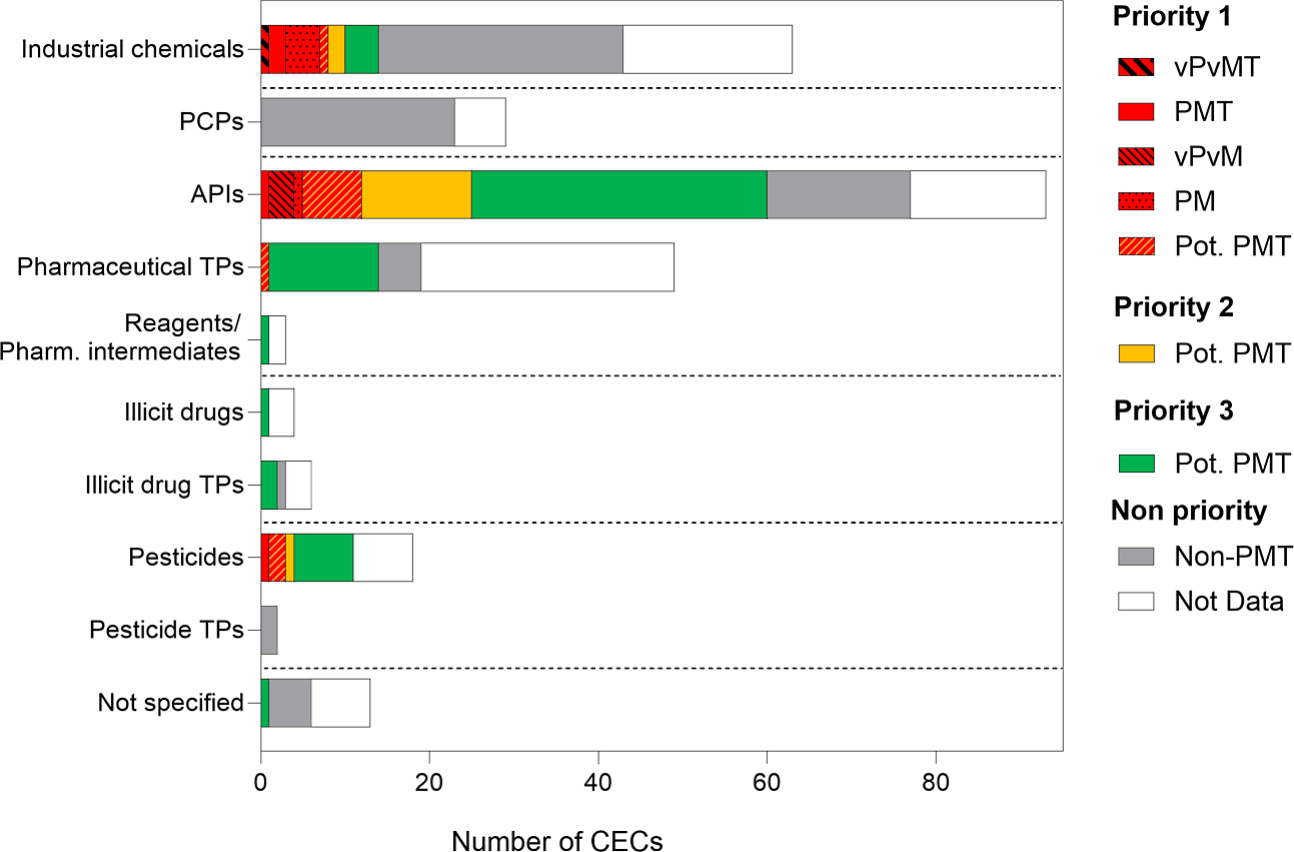
Stacked bar chart illustrating the number of tentatively identified substances according to their use, along with PMT classification, and priority levels.

First, substances are classified according to their persistence. Within experimental data, *t*_1/2_ is available for only 6% of the total tentatively identified substances. This result highlights the crucial role of QSAR models in the persistence assessment. However, 34% (102 substances) cannot be classified according to their persistence due to the lack or inconsistency of data. Among these, pharmaceutical TPs and industrial chemicals accounted for about 60%. These results emphasize that the assessment of persistence remains the primary drawback in the classification of substances according to P-M-T criteria and that, for pharmaceutical TPs and industrial chemicals, additional efforts should be made to fulfill the data gap. Based on available data, 2% of the substances are classified as vP, 3% as P, 28% as Not P, and 34% are Pot. P. Second, the 118 substances categorized as belonging to distinct classes of persistence (vP, P, P, P) are classified according to their mobility. Among these, 54% of the substances are classified as vM, 25% as M, 9% as Potential M/vM, and 11% as Not M. Finally, substances were classified according to their toxicity. Hence, 30% of the substances are classified as T, while 69% are categorized as Pot.T. Only a minimal percentage (1%) is classified as Not Toxic (Not T). Among substances classified as T, 8 exhibit endocrine-disrupting properties, 27 substances are classified as carcinogenic, mutagenic, or reprotoxic (CMR), 4 present specific target organ toxicity following repeated exposure (STOT RE) according to Annex XIII of the REACH Regulation, and 3 show a NOEC value below 0.01 mg/L.

According to the classification through the six classes that take into account the P, M and T criteria (Prioritization Strategy Section) all together and the appearance in selected lists (Commission Implementing Decision (EU) 2022/1307, Proposal for amending Directive 2000/60/EC, Directive 2006/118/EC and Directive 2008/105/EC,^38^ and REACH-SVHC list), a total of 103 substances comply with one of the defined PMT categories. Hence, 1 CEC is classified as vPvMT (benzotriazole), 5 as PMT, 4 as vPvM, 4 as PM, and 94 as Potential PMT.

Finally, levels of Priority from 1 to 3 are assigned to each of the 103 classified substances (Table S5), resulting in (i) 25 substances with a level of Priority 1, (ii) 18 substances with a level of Priority 2, and (iii) 60 substances with a level of Priority 3. Notably, 51 substances classified as Potential PMT are not registered under REACH. Instead of basing the process on a predefined list of PMT substances and then verifying their presence in the sample, our workflow was designed to first detect the widest possible range of contaminants and classify them according to PMT criteria. To our knowledge, this approach addresses an existing gap in current assessments, which may overlook potentially relevant PMT/vPvM substances that are not yet included in the REACH regulation. This strategy including SSA to prioritize poorly investigated substances is also proposed in the updated NORMAN prioritization scheme.^39^ In addition, the data generated in this study could provide exposure evidence useful for prioritization strategies or an EU workflow aiming to select candidates for Priority Substances or EU Watch lists.

The pharmaceutical TPs have been the subclass of substances with the greatest lack of information. Therefore, efforts should be made to obtain more high-quality persistence data on substances such as pharmaceutical TPs, as most of them are unregulated in the European Union and generally not included in routine chemical risk or hazard assessment.^40^ Moreover, only 13% of PMT substances appeared in European Water Framework Directive legislation or a list of substances of very high concern.

Some of the prioritized substances (Priority 1–3) remain scarcely investigated in the context of environmental monitoring studies. For example, the pharmaceutical celecoxib, despite being one of the most widely used anti-inflammatory drugs, has received limited attention in aquatic ecosystem research.^41,42^ Regarding pharmaceutical TPs, pantoprazole sulfide and *N*-acetylsulfapyridine (TPs of the widely prescribed pantoprazole and sulfapyridine, respectively) are almost never considered in environmental investigations.^43,44^ On the other hand, tapentadol-*O*-sulfate and *o*-desarylranolazine have only recently been detected in wastewater.^45^ Furthermore, to the best of the authors’ knowledge, rabeprazole sulfide (TP of the proton pump inhibitor rabeprazole) as well as ranolazine and its TPs (*o*-sesarylranolazine and *N*-(2,6-dimethylphenyl)-1-piperazineacetamide) have not been previously reported in the environment.

Similarly, most Priority level 1 and 2 industrial additives are produced in substantial quantities within the European Economic Area (Registered substances, ECHA); however, they have attracted little scientific scrutiny. One example is 1,3-di-*o*-tolylguanidine (DTG), a chemical raw material extensively used in industry, medicine, and other fields,^46^ whose environmental occurrence has been scarcely investigated.^47^ Another case is the additive 3-aminomethyl-3,5,5-trimethylcyclohexylamine (IPDA), a compound widely used in the production of adhesives, sealants, coatings, fillers, putties, plasters, and modeling clay. IPDA was first reported in environmental samples by Schultze et al. (2019),^36^ suggesting its status as a novel compound in the environment. A further notable example is the ultrashort-chain PFAS bis­(trifluoromethylsulfonyl)­imide, for which environmental occurrence data remains extremely limited.^48^

#### Confirmation

Confirmation with analytical standards was successfully performed for 38 of the 40 PMT substances classified as Priority 1 and 2 in the LC-HRMS screening. Oxazepam and lormetazepam were excluded due to the lack of available standards. The comparison of precursor ion mass accuracy (<5 ppm), chromatographic profiles (*t*_R_ ± 0.1 min), and MS/MS spectra (identification of at least 2 characteristic fragment ions) provided by LC-HRMS is conclusive enough to confirm the occurrence of most of these substances in the surface water sample.

As an example, Figure 1 (see the confirmation step) shows the chromatographic peak and MS/MS­(QTOF) spectrum of the sitagliptin analytical standard. As can be observed, the *t*_R_ (11.12 min) and the fragment pattern (*m*/*z* = 174.052, 193.069, 235.079) of sitagliptin agree very well with the molecular ion identified in the sample (11.08 min and *m*/*z* = 174.052, 193.069, 235.081).

In other cases, the high background in the MS/MS spectra is a challenge for the confirmation of the substances. Figure 6 shows the cases of amantadine and benzotriazole. For both compounds, the interpretation of MS/MS­(QTOF) spectra can be ambiguous due to the low intensity of ions or *m*/*z* values <100. For amantadine, the great variation in the abundance ratio of the most characteristic MS/MS ions between the sample and the analytical standard can be explained by the low intensity (S/N = 73) of the precursor ion ([M + H]^+^, *m*/*z* = 152.143) in the sample. Generally, low S/N ratios are due to two possible reasons: (i) low compound concentration or (ii) the lack of sensitivity (concentrations near the detection limit). The case of benzotriazole is different. Here, the S/N ratio of the precursor ion is high (S/N = 991), but the *m*/*z* values of the most characteristic molecular fragments are <100 (65.038 and 72.936). In the area of the MS/MS spectrum between 50 and 100 *m*/*z*, ions could overlap with background interferences, giving poor accurate mass measurements and alteration of their isotopic distribution.^49^ Such examples demonstrate how fragmentation data are essential for confirmation studies.

**Figure 6:**
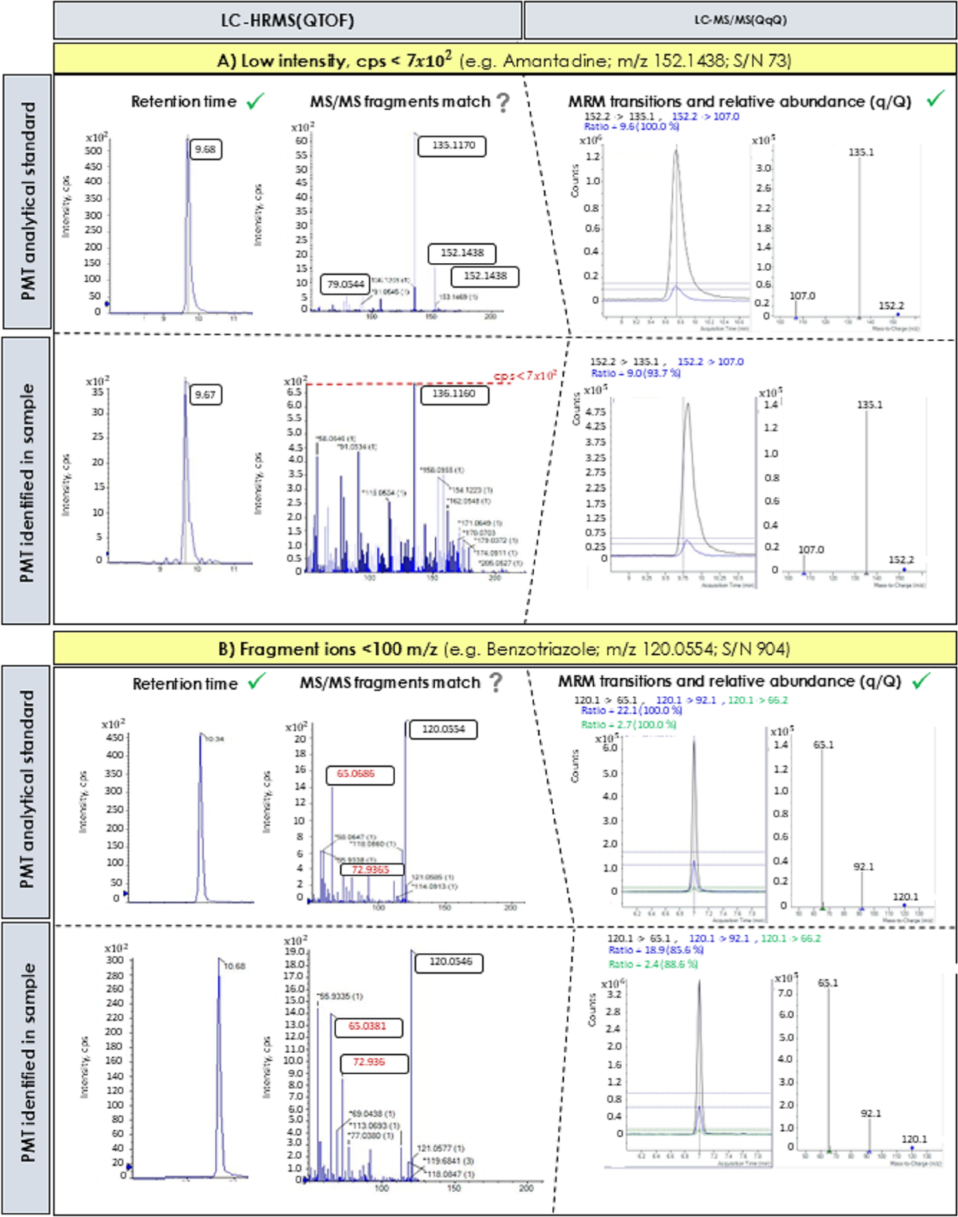
Unequivocal confirmation of suspect PMT substances: amantadine and benzotriazole. (A) The low signal-to-noise (S/N) of amantadine in the sample hinders the interpretation of the MS/MS spectrum obtained by LC-HRMS. (B) The low *m*/*z* values of fragment ions in the MS/MS spectrum obtained by LC-HRMS hinder the confirmation.

In this work, an additional MS/MS approach was required to corroborate the identification of those substances for which similarities of QTOF-MS/MS patterns between the sample and standard were ambiguous: codeine, diazepam, chloramphenicol, and furosemide. These substances were confirmed by using analytical standards by LC–MS/MS with a QqQ analyzer. A targeted MS/MS method was developed for these analytical standards including the three substances from the target screening (1,3-di-*o*-tolylguanidine, 3-aminomethyl-3,5,5-trimethylcyclohexylamine, and triethyl phosphate), and product ions and CE were optimized to select the most characteristic multiple reaction monitoring (MRM) transitions for each analyte. The information about MRM transitions and optimized CE is summarized in Supporting Information (Table S6). After the confirmation analysis by checking MRM transitions with their relative abundances, 4 substances are unequivocally confirmed in the sample. These results underscore the usefulness of combining MS/MS (QqQ) with HRMS to obtain reliable fragmentation data. Approximately 90% of suspect positives are unequivocally confirmed: 28 substances were verified using LC-HRMS with corresponding analytical standards, and 4 substances were confirmed after an in-depth investigation of their MS/MS spectra by LC–MS/MS­(QqQ). False positives are predominantly associated with confidence level 3, accounting for 5 out of 6 cases. All substances with the highest confidence level were confirmed, supporting the relevance of using detection-based confidence levels as a basis for candidates. Figure 7 shows a classification of Priority 1 substances including the confidence level, PMT classification, SPE cartridge, LC columns, and confirmation by LC-QTOF or LC–MS/MS. Other methodological indicators (*m*/*z*, library score, etc.) of Priority 1 and 2 substances (nr 43) are detailed in Table S7. Among classified PMTs, Priority 1 and 2 substances should be recognized as highly relevant for monitoring purposes. In relation to the actual legislative framework, about 72% of the confirmed PMTs are not included in the European Water Framework Directive and 42% are not registered under the REACH regulation.

**Figure 7:**
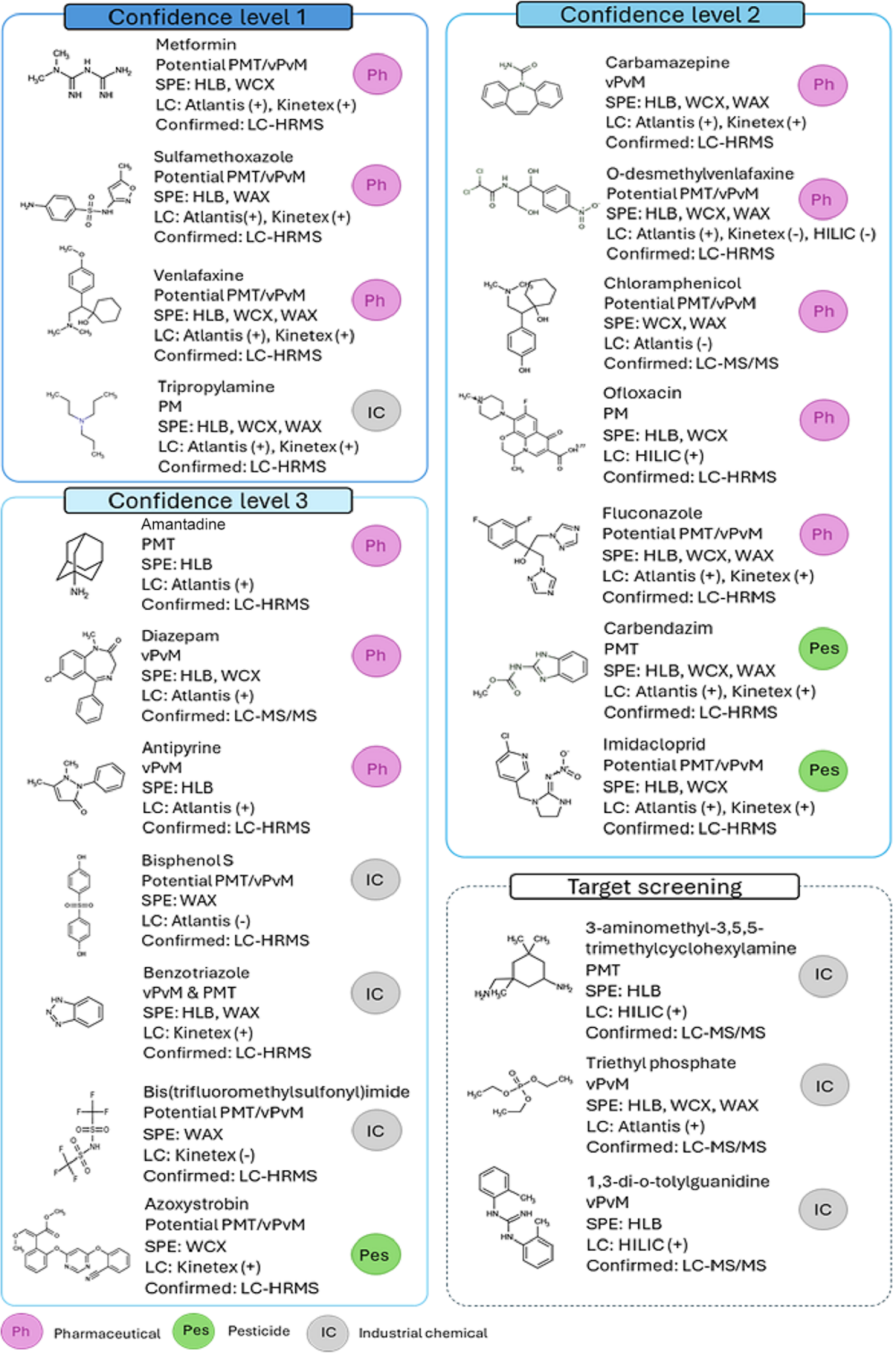
Confirmed Priority 1 substances, including name, class, PMT classification, and the specific SPE cartridge and LC column by which each substance was detected in the SSA, as well as confirmation by LC-MS/MS (qQq) or LC-HRMS (QTOF).

In addition, we assessed the suitability of the RTI system under our analytical conditions. Approaches based on the t_R_ provide additional information in screening analyses and can help reduce false positives.^50^ For this purpose, the 18 RTI calibrants were analyzed by LC-HRMS. For 4 of them (guanylurea, amitrole, histamine, and chlormequat), the elution times are below t_0_ (between 0.91 and 1.1 min) under our analytical conditions. Hence, RTI calibration curves are obtained exclusively considering the 14 remaining 14 calibrants that are retained under general RPLC conditions (using an Atlantis dC18 column and a mobile phase of MeOH/H_2_O with 0.1% formic acid). Since very polar compounds exhibit poor retention in a typical C18 column, these approaches are still not suitable for them.^51^ This represents a limitation of the system, as the first RTI calibrant had a *t*_R_ of 3.7 min, and most polar compounds cannot be included.

Matrix interferences commonly occurring in preconcentrated samples can hamper the performance of the RTI system (modification of *t*_R_, signal suppression of calibrants, etc.). To evaluate the potential impact of ME, the 14 RTI calibrants were analyzed in the different SPE extracts, and the results were compared to those obtained in the MeOH/water 10:90% (v/v) medium (Table S3). Thirteen calibrants are detected in HLB SPE fortified extracts, 11 in WCX, and 12 in WAX. The t_R_ of the calibrants in the SPE extracts is similar, and therefore, the calibration equations are also comparable (Figure S2), indicating that in this case, ME is not significant for the RTI calibration curves.

Therefore, the RTI system was used for some of the substances selected for confirmation (Table S7). Hence, among the 43 prioritized substances, the RTI system was applied to a selected subgroup that met the following criteria: (i) retention on the Atlantis dC18 column, (ii) ionization in ESI + mode, and (iii) availability of an analytical standard in our laboratory. According to these criteria, 23 substances are finally selected. Of these, 17 showed predicted RTIs in agreement with the confirmation results; 5 had *t*_R_ values that do not fit with the RTI model but nonetheless were confirmed by analytical standards. In contrast, flucytosine is identified as a false positive. Despite the limited number of calibrants detected under our analytical conditions, the RTI system demonstrated its utility as a supportive tool for SSAs, providing an additional confidence level in substance identification.

#### Future Perspectives for the Development of a Targeted Multiresidue Quantification Method

The SSA, when combined with the prioritization strategy applied in this study, provides valuable insights into the environmental occurrence of PMT substances. This integrated methodology represents a foundational step toward the development of monitoring strategies, which must ultimately be supported by using robust and targeted quantification methods.^52^ For such methods, and in particular for multiresidue approaches based on LC–MS/MS, systematic optimization of instrumental parameters, chromatographic separations, and extraction conditions is essential to ensure the maximum response and reproducibility for the analytes of interest. In this sense, a key objective of the workflow proposed in this study is to leverage the analytical information generated through SSA to streamline future optimization of targeted multiresidue methods for quantifying PMT substances in water samples. To find the most suitable response of confirmed PMT substances, the following key parameters (Table S7) have been evaluated: (i) elution behavior (chromatographic columns and *t*_R_); (ii) ionization response (ESI+/ESI- and S/N of molecular ions); and (iii) extraction capacity (SPE protocols). This information will guide the selection of optimal separation, ionization, and extraction conditions, facilitating the subsequent optimization and validation of the target analytical method in the next phase of this research.

The LC separation data presented in Table S7 show the identification of PMT substances under RP conditions using Atlantis dC18 and Kinetex F5 columns, as well as under HILIC conditions using the SeQuant-ZIC column, along with their t_R_. Of the 36 confirmed PMT substances, the majority are identified under RP conditions, with 4 substances confirmed through the HILIC mechanism. Between the RP columns, the Atlantis dC18 demonstrated superior performance, enabling the detection of 29 PMT substances compared to 22 with the Kinetex F5. Additionally, the Atlantis dC18 column provides better retention capabilities with only 1 PMT substance eluting in dead time (*t*_0_ < 1.65 min) compared to the F5 column with 3 PMT substances. In addition, substances such as 4-aminoantipyrine, atenolol, carbendazim, metronidazole, sulpiride, and tripropylamine exhibit markedly improved retention between 7.0 and 10.1 min in the Atlantis dC18 column, compared to earlier elution (2.21–3.83 min) when using the Kinetex F5 column. Early elution at the beginning of the chromatographic gradient may increase the risk of matrix interference coelution, potentially compromising peak resolution and/or sensitivity. Based on the obtained results, we selected the Atlantis dC18 columns for a more exhaustive optimization of the chromatographic separation, including the evaluation of column temperatures, flow rates, and organic solvent compositions.

The optimal ionization mode (ESI + or ESI−) for the confirmed PMT substances can also be determined using the S/N ratio provided by SSA, under the same sample treatment. As shown in Table S7, the majority of PMT substances are identified under positive ionization conditions, with 28 substances detected as [M + H]^+^ molecular ions, compared to 6 detected as [M – H]^−^. Only valsartan and O–desmethylvenlafaxine are detected in both modes, with similar S/N ratios in ESI+ (Table S2).

Finally, regarding the best SPE protocols, the use of HLB and WCX cartridges is more effective for extracting most of the PMT substances (Table S7). Specifically, 12 PMT substances are detected in HLB extracts, 6 substances are identified in WCX extracts, and 16 are identified in both. Only two substances (4,4′-sulfonylbisphenol and bis­(trifluoromethylsulfonyl)­imide) are identified following extraction with WAX cartridges. Given the limited effectiveness of WAX, its use has been discarded from further method development. Instead, future studies will explore different SPE strategies employing HLB and WCX cartridges, either independently or in tandem, to evaluate recoveries of PMT substances and optimize the extraction efficiency.

In summary, the use of SSA not only facilitates the detection of a broad spectrum of PMT substances but also provides critical information to guide and support the development of a robust multiresidue targeted analytical method for monitoring the occurrence of the 35 PMT substances in environmental water matrices.

## Conclusions

The integrated workflow designed in this study, which combines SSA with a prioritization strategy, represents an effective and scalable approach to enhance the current state of knowledge on the occurrence of PMT/vPvM substances in the environment. The comparative evaluation and combination of different SPE sorbents with multiple LC methods for PMT detection allowed the successful confirmation of 35 PMT substances in a surface water sample affected by the discharge of WWTPs. Importantly, the proposed methodological framework presented in this study can be easily adapted to a wide range of environmental scenarios, providing valuable support for future monitoring initiatives.

From an analytical perspective, key conclusions to guide further related studies can be summarized as follows:

- Sample pretreatment. Preliminary evaluation of ME at different CFs allows us to simplify and strengthen the step of data processing and filtering. The combination of multiple SPE protocols enables the extraction of a broad spectrum of substances, effectively bridging the gap between permanently charged and ionizable substances.
- Analysis by LC-HRMS. The HILIC separation mechanism enables the detection of the greatest number of highly hydrophilic suspect substances. In contrast, the RPLC separation mechanism allows the detection of the highest total number of substances.
- Confirmation study. The most confirmed PMT substances were those retained by Atlantis dC18 due to its robustness and versatility. The combination of HRMS (QTOF analyzer) with MS/MS spectrometry (QqQ analyzer) is critical for the confirmation using analytical standards. Despite the limitations of the RTI system, its use still provides useful data that offers an additional level of confidence and helps streamline the confirmation step in SSA.

The integrated workflow using SSA enables the tentative identification of substances whose occurrence in the environment is poorly reported or unknown, thereby filling critical gaps in chemical exposure data. Finally, the authors emphasize that a significant proportion of substances classified as potential PMT/vPvM (60 out of 103) were not selected for confirmation due to the lack of toxicity experimental data. Most of these potential PMT/vPvM substances are pharmaceutical TPs. Future research efforts should therefore focus on generating high-quality data on persistence and toxicity, particularly for TPs, to enable comprehensive risk assessments and the effective monitoring of PMT substances. This is especially important given that most of these substances remain unregulated within the European Union and, consequently, are often excluded from routine chemical risk or hazard assessments.

## Supplementary Material





## Notes

Conditions of mass spectrometry in tandem (MS/MS) using a LC (HPLC 1200 Agilent series, Palo Alto, CA, USA) coupled to an Agilent 6495 triple quadrupole mass spectrometer (LC–MS/MS­(QqQ)); solid-phase extraction protocols; composition of the mobile phases for Atlantis dC18, Kinetex F5, and ZIC-HILIC; relationship between the log *D*_ow_ and the retention time of the substances tentatively detected (Figure S1); and RTI (ESI+) calibration curves (Figure S2) (PDF)
List of standards (Table S1); screening results (Table S2); RTI index (Table S3); mobile phases polar substances (Table S4); information about MRM transitions (Table S5), prioritization results (Table S6); and substances selected for confirmation (Table S7) (XLSX)
