# Supplementary material for: Suspect Screening and Prioritization as an Analytical Strategy for the Identification of Persistent, Mobile, and Toxic (PMT) Substances in Surface Water
Source: Anal Chem. 2026 Feb 19;98(8):5923–37. doi: 10.1021/acs.analchem.5c04907 (PMC12961641; doi:10.1021/acs.analchem.5c04907)
Supplement: Supplementary file 1 [file ac5c04907_si_001.pdf]

Suspect screening and prioritization as analytical strategy for the identification of persistent, mobile and toxic (PMT) substances in surface water.

Lesly Ayala Cabana [*] [a,b], Alejandra Arcas [b], Isabel López-Heras [b], Ana de Santiago Martín [b], Raffaella Meffe [b]

[a] University of Alcalá, Geology, Geography and Environment Department, Faculty of Sciences, External Campus, Ctra. A-II km 33.6, 28871 Alcalá de Henares, Madrid, Spain
[b] IMDEA Water, Avda Punto Com, 2, 28805 Alcalá de Henares, Madrid, Spain
*corresponding author: lesly.ayala@edu.uah.es

Table of contents

SI 1. Materials and Methods

SI 1.1. Conditions of mass spectrometry in tandem (MS/MS) using a LC (HPLC 1200 Agilent series, Palo Alto, CA, USA) coupled to an Agilent 6495 triple quadrupole mass spectrometer (LC-MS/MS(QqQ)) S2

SI 1.2. Solid phase extraction protocols S2

SI 1.3. Composition of the mobile phases for Atlantis dC18 and Kinetex in positive and negative ionization mode, and other separation parameters S2

SI 1.4. Composition of the mobile phases for ZIC-HILIC in positive and negative ionization mode and other separation parameters S3

SI 2. Results

Figure S 1. Relationship between the Log D_{ow} and the retention time of the substances tentatively detected by a) Atlantis dC18, and b) Kinetex F5, c) Atlantis dC18-HFBA and d) HILIC at pH 7. .... S3

Figure S 2. RTI (ESI+) calibration curves from: a) RTI standard prepared in MeOH:water 10:90% (v/v) medium, and from fortified extract after SPE using b) HLB cartridge, c) WAX cartridge, or d) WCX cartridge. The RTI platform was used to obtain the calibration curves and corresponding equations (http://rti.chem.uoa.gr/) (Aalizadeh et al. 2021). S4

SI 1. Materials and Method

SI 1.1. Conditions of mass spectrometry in tandem (MS/MS) using a LC (HPLC 1200 Agilent series, Palo Alto, CA, USA) coupled to an Agilent 6495 triple quadrupole mass spectrometer (LC-MS/MS(QqQ))

The experiments to select the MRM transitions of each compound were performed in the FIA (Flow Injection Analysis) mode using a dead volume connection between the injector and ESI source. First, standard solutions of 1 mg/L containing all chemicals were analysed in the full-scan mode (m/z 50–1000) to identify the precursor ions. Next, a 0.1 mg/L solution was used to identify the product ions by applying four collision energies (CE): 10, 20, 30 and 40 V. ESI parameters of MS/MS analyses: the drying gas temperature was set at 250 °C and 13 L/min; sheath gas at 350 °C and 11 L/min; nebulizer pressure at 45 psi; capillary voltage at 4000 V (ESI +) / −3000 V (ESI-), and chromatographic conditions employed in SSA.

SI 1.2. Solid phase extraction protocols

The protocol used for OASIS HLB (200 mg, 6 cc) cartridges was as follows: 200 mL of sample was passed through the cartridge previously conditioned with 6 mL of MeOH and 8 mL of ultrapure water. After loading the sample, the cartridge was rinsed with 10 mL of ultrapure water and dried under vacuum (5 bar) for a few seconds to eliminate residual water. Analytes were eluted with two aliquots of 6 mL of MeOH. For OASIS WAX and WCX, the SPE protocol was subjected to modifications in conditioning, wash, and elution steps. The OASIS WAX (150 mg, 6 cc) cartridges were previously conditioned with 6 mL of 2% (v/v) formic acid in MeOH and 8 mL of ultrapure water. The cartridges were washed with 10 mL of 2% (v/v) formic acid in ultrapure water, and the elution was performed with 6 mL of MeOH, followed by 6 mL of 5% (v/v) NH_4OH in MeOH. In the case of OASIS WCX (150 mg, 6cc) cartridges, the conditioning was done with 6 mL of 5% (v/v) NH_4OH in MeOH and 8 mL of ultrapure water. The cartridges were washed with 10 mL of 5% (v/v) NH_4OH in ultrapure water and the elution was performed with 6 mL of MeOH, followed by 6 mL of 2% (v/v) formic acid in MeOH.

SI 1.3. Composition of the mobile phases for Atlantis dC18 and Kinetex in positive and negative ionization mode, and other separation parameters

The composition of the mobile phases for Atlantis dC18 were: (i) ultrapure water with 0.1% formic acid as eluent A and MeOH with 0.1% formic acid as eluent B for ESI+; (ii) water with 5 mM ammonium acetate (pH 5.8) as eluent A and MeOH:water 90:10% (v/v) with 5 mM ammonium acetate (pH 5.8) as eluent B for ESI-. The mobile phases used for the Kinetex F5 column were the same as those for Atlantis dC18 column, with the exception that ACN replaced MeOH as the organic solvent. A linear binary gradient was applied for both columns and ionization modes: from 2% to 100% of eluent B over 20 min. The 100% of eluent B condition was maintained for 5 min and after the analysis, the columns were re-equilibrated for 5 min using the initial solvent composition. The flow rate and temperature were set at 0.4 mL/min and 40 °C for Atlantis dC18, and 0.3 mL/min and 50 °C for Kinetex F5, respectively. The injection volume was set to 10 µL.

SI 1.4. Composition of the mobile phases for ZIC-HILIC in positive and negative ionization mode and other separation parameters

The composition of mobile phases was as follows: (i) ACN:water with 5mM ammonium formate pH 3.2), 90:10% (v/v), as eluent A and ACN:water with 5mM HCOONH4 (pH 3.2), 10:90% (v/v), as eluent B for ESI+; and (ii) ACN:water with 5mM ammonium acetate, (pH 5.8), 90:10% (v/v), as eluent A and ACN:water with 5mM ammonium acetate (pH 5.8), 10:90% (v/v), as eluent B for ESI-. For both ionization modes, the same separation gradient was used: the initial percentage of eluent A was 90% (held for 2.5 min), which was linearly decreased to 50% in 15 min, followed by a 2.5 min isocratic period and then returned to initial conditions during 3.5 min. The column temperature was maintained at 40 °C, the flow rate was 0.4 mL/min, and the injection volume was set to 10 μL.

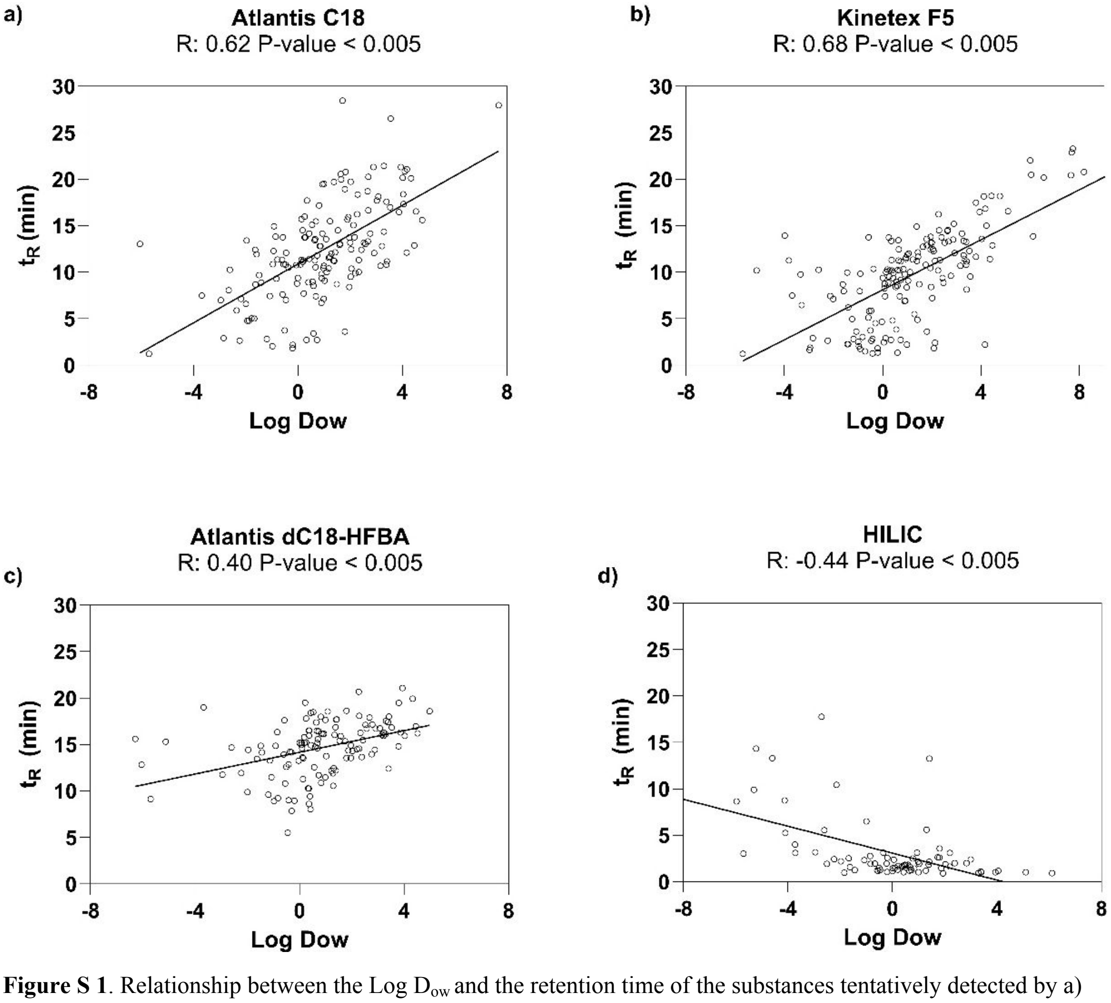

Figure S 1. Relationship between the Log D_{ow} and the retention time of the substances tentatively detected by a) Atlantis dC18, and b) Kinetex F5, c) Atlantis dC18-HFBA and d) HILIC at pH 7.

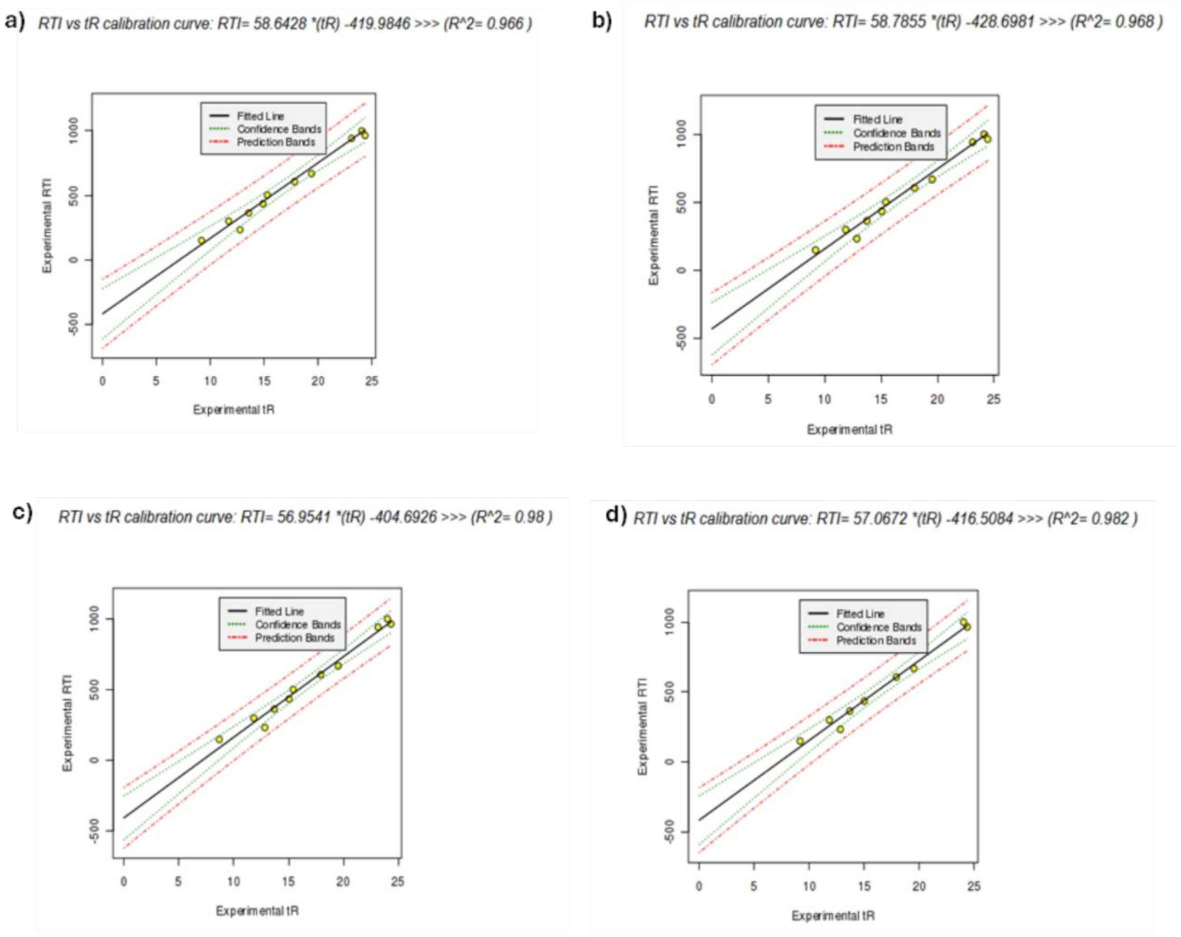

Figure S 2. RTI (ESI+) calibration curves from: a) RTI standard prepared in MeOH:water 10:90% (v/v) medium, and from fortified extract after SPE using b) HLB cartridge, c) WAX cartridge, or d) WCX cartridge. The RTI platform was used to obtain the calibration curves and corresponding equations (http://rti.chem.uoa.gr/) (Aalizadeh et al. 2021).
